# Supplementary material for: Eph receptor tyrosine kinases are functional entry receptors for murine gammaherpesvirus 68
Source: PLoS Pathog. 2025 Oct 21;21(10):e1013263. doi: 10.1371/journal.ppat.1013263 (PMC12561925; doi:10.1371/journal.ppat.1013263)
Supplement: Attachment — Submitted filename: Response_to_Reviewers_AG_2025.docx [file ppat.1013263.s007.docx]

*Manuscript PPATHOGENS-D-25-01356*

**Response to Reviewers**

We sincerely thank the reviewers for their thorough and thoughtful evaluation of our manuscript. Their constructive feedback and insightful suggestions led us to enhance the clarity and quality of the study. In response to these comments, we performed additional immunoprecipitation experiments confirming the binding of KSHV gH/gL to murine EphA2 and EphA4 (S1F Fig), provided primary infection rates (S2 Fig, S2 Table), and strengthened and clarified statements regarding our observations in the results and discussion. The document with changes highlighted/ tracked are included in the submission.

**Reviewers' Comments:**

Reviewer's Responses to Questions

**Part I - Summary**
Please use this section to discuss strengths/weaknesses of study, novelty/significance, general execution and scholarship.

Reviewer #1: Grosskopf et al report here that the Eph receptor tyrosine kinases function as receptors for murine gammaherpevirus 68. The EphR TKs were previously shown to serve as receptors for EBV, KSHV and RRV through binding the conserved gH/gL glycoproteins, findings that underpin the significance of this new report. Vaccination and immune protection from gammaherpesvirus infection and subsequent diseases is an important goal, and therefore demonstration that the mouse model system displays remarkable conservation of the critical entry interaction is very important to the field and to future studies.
This report is thorough and straightforward, making use of in silico comparative studies, screening of multiple EphR family members, use of purified viral glycoprotein complexes, analysis of MHV68 infection and neutralization with purified complexes and domains, and demonstration of the sufficiency of the EphR A4 and B3 for infection of human cells. The conclusions of this report are fully supported, and indicate the utility of the conserved MHV68 infection entry system for modeling and testing gammaherpesvirus entry.

Reviewer #2: Prior research has identified Eph receptors as key entry receptors engaged by the gH/gL complex of human gammaherpesviruses. This study by Grosskopf et al. is the first to demonstrate Eph receptors, specifically EphA4 and EphB3, as functional entry receptors for murine gammaherpesvirus 68 (MHV68). The manuscript presents multiple lines of evidence supporting their conclusion: interactions of MHV68 gH/gL with EphA4 and EphB3, inhibition of infection in permissive cell types by soluble EphA4 and B3, and the ability of ectopically expressed EphA4 and B3 to render otherwise non-permissive human Raji B cells susceptible to MHV68 infection. These experiments include another Eph receptor, EphA2 as a negative control for specificity. Guided by modeling and alignment with the existing structure of KSHV gH/gL-EphA2 complex, the authors experimentally demonstrated that the interaction between MHV68 gH/gL and EphA4 involves specific residues at domain I of MHV68 gH. This confirms evolutionary conservation of entry mechanisms. Overall, this study represents a significant advancement in gammaherpesvirus research and establishes a strong foundation for using MHV68 as an in vivo model system for pathogenesis and vaccine development. The manuscript is well written, clearly presented with solid experimental data, and easy to follow. Only a few clarifications are needed to further enhance the clarify of the study.

Reviewer #3: Although entry receptors for EBV and KSHV, including Eph family members, have been well-described, entry receptors for the related murine virus MHV68 have not been previously determined. Work in this manuscript directly tests the ability Eph family members to interact with MHV68 glycoproteins and serve as entry receptors. Through a series of beautiful experiments, the authors identify EphA4 and EphB3 as direct interaction partners of the MHV68 gH/gL complex, and further demonstrate that expression of EphA4 and B3 facilitates infection of multiple cell types from both humans and mice. This is outstanding, well-written report with carefully controlled experiments and clear conclusions. These exciting results provide a large step forward for the field and provide important additional evidence for the evolutionary conservation of MHV68 with other human and primate herpesviruses.

**Part II – Major Issues: Key Experiments Required for Acceptance**
Please use this section to detail the key new experiments or modifications of existing experiments that should be absolutely required to validate study conclusions.

Generally, there should be no more than 3 such required experiments or major modifications for a "Major Revision" recommendation. If more than 3 experiments are necessary to validate the study conclusions, then you are encouraged to recommend "Reject".

Reviewer #1: no major issues

Reviewer #2: None

Reviewer #3: NONE

**Part III – Minor Issues: Editorial and Data Presentation Modifications**
Please use this section for editorial suggestions as well as relatively minor modifications of existing data that would enhance clarity.

**Reviewer #1: Minor issues that could be discussed:**
1) In figure 2, it appears that soluble EphA2 trends toward enhancement of infection. It would be informative to learn whether this is considered to be technical or specific.

We agree with the reviewer that the slight but consistently observed increase in infection upon sEphA2 treatment is an interesting phenotype. However, since this increase did not reach statistical significance, we did not pursue in-depth characterization. Given the experimental setup, we cannot exclude potential effects of soluble Eph receptors on membrane-bound ephrin ligands. In the future, a more detailed analysis of Eph and ephrin expression in the cell types used, along with complementary blocking experiments, may help clarify the technical or biological relevance of this observation. We added a statement to address this observation to the manuscript (lines 338 ff.).

2) As shown in figure 5, antisera depletion of gH/gL specific antibodies does decrease neutralization of infection, but only to approximately 50% as the authors point out. Is this due to the capacity for herpesviruses to use multiple means of entry, incomplete depletion of gH/gL antibodies specific to epitopes not shared with the purified gH/gL reagent, other? Some discussion of this is useful in thoughtful consideration of focusing on neutralizing gH/gL in gammaherpesvirus vaccination.

We thank the reviewer for highlighting these important considerations. We indeed observe incomplete antibody depletion, consistent with the partial reduction in neutralization activity (see Fig 5 and S5 Fig). Technical factors may contribute to these observations, including subtle differences in reagents used for ELISA detection versus depletion protocols (fusion protein vs co-expressed gH/gL), binding kinetics, antigen orientation as well as potential effects of protein purification on gH/gL folding or antigen accessibility. Additionally, lower-affinity antibodies may bind less efficiently to the magnetic beads, which potentially present antigen at lower density compared to ELISA plates. To address this, we have added a brief discussion highlighting these potential confounding factors (lines 407 ff.).

**Reviewer #2:**

1. Fig. 2. Are all cell types equally susceptible to MHV68 infection? In other words, what are the percentage of GFP+ cells in the PBS controls? This information should be included.

We thank the reviewer for addressing this omittance. We now provide infection as raw GFP percentages as supplementary figure 2 (S2A-D Fig) and S2 Table.

2. LINE 180: Typically, lower EC50 values indicates higher affinity. Therefore, the higher EC50 observed for mEphA4 is somewhat counterintuitive. The authors should clarify the interpretation of EC50 and maximum OD values in the context of receptor and gH/gL interactions.

Thank you for this insightful comment. We agree that the higher EC50 observed for mEphA4 suggests a lower apparent affinity or slower binding kinetics under these assay conditions, while the higher maximum OD reflects greater overall binding capacity at saturation. We have added a clarification on the interpretation of OD versus EC50 to the discussion (lines 369 ff.). The precise mechanisms underlying these differences—such as variations in complex stability, additional interaction sites on mEphA4, or differences in receptor valency—represent interesting avenues for future investigation.

3. Fig. 3D. Only fold change is displayed. The authors should indicate what a >20-fold increases correspond to regarding the percentage of GFP+ cells.

We added the range of infection for empty vector controls to the figure legend in the manuscript (line 701) and provide primary infection data as S2 Table.

4. Fig. 5B. The authors should indicate whether undiluted or diluted serum was used. If diluted serum was used for ELISA, include the dilution factor.

We apologize for missing this important information. The dilution factor (1:80) was

added in the figure legend (line 732).

5. Does KSHV gH/gL complex interact with murine EpHA2 and A4?

We thank the reviewer for addressing this important consideration for the use of MHV68 chimeras in vivo studies. We added a comparative analysis of KSHV gH/gL interaction with murine EphA2 and EphA4 as part of S1F Fig (lines 166 ff.). Binding of KSHV gH/gL to murine EphA2 and EphA4 recapitulates binding to human Ephs.

**Reviewer #3: MINOR**
1. Fig 2C – 2G. Figures indicate percent GFP relative to control, but give no indication of total GFP+ cells (ie, percent cells infected). This should be stated clearly (or raw data included), perhaps as a supplementary figure or table.

We thank the reviewer for addressing this omittance. We now provide infection as raw GFP percentages as supplementary figure 2 (S2A-D Fig) and S2 Table.

2. MHV68 ORF59-GFP should be described in slightly more detail in the main text to indicate the nature of the GFP expression and the role of ORF59.

We expanded on the description of ORF59 and ORF59-GFP expression to the manuscript (lines 194 ff.).
